# The synthetic opioid fentanyl increases HIV replication in macrophages

**DOI:** 10.1371/journal.pone.0298341

**Published:** 2025-02-27

**Authors:** Janani Madhuravasal Krishnan, Ling Kong, Heidi L. Meeds, Krishna M. Roskin, Mario Medvedovic, Kenneth E. Sherman, Jason T. Blackard

**Affiliations:** 1 Division of Digestive Diseases, Department of Internal Medicine, University of Cincinnati College of Medicine, Cincinnati, OH, United States of America; 2 Division of Biomedical Informatics, Cincinnati Children’s Hospital Medical Center, Cincinnati, OH, United States of America; 3 Department of Pediatrics, University of Cincinnati College of Medicine, Cincinnati, OH, United States of America; 4 Department of Environmental & Public Health Sciences, University of Cincinnati College of Medicine, Cincinnati, OH, United States of America; 5 Center for Addiction Research, University of Cincinnati College of Medicine, Cincinnati, OH, United States of America; Fisheries and Oceans Canada, CANADA

## Abstract

**Background:**

The illicit use of synthetic opioids such as fentanyl has led to a serious public health crisis in the US. People with opioid use disorder are more likely to contract infections such as HIV and viral hepatitis and experience more severe disease. While several drugs of abuse are known to enhance viral replication and suppress immunologic responses, the effects of synthetic opioids on HIV pathogenesis have not been investigated thoroughly. Thus, we examined the impact of fentanyl on HIV replication and chemokine receptor expression in the U937 cell line and monocyte-derived macrophages (MDMs).

**Methods:**

U937 cells were exposed to varying concentrations of fentanyl. Expression levels of the CXCR4 and CCR5 chemokine receptors were measured in cell lysates. HIV p24 antigen was quantified in culture supernatants by ELISA, and HIV proviral DNA was quantified in cells using SYBR real-time PCR targeting the *pol* gene. RNAseq was performed to characterize cellular gene regulation in the presence of fentanyl.

**Results:**

Fentanyl induced HIV p24 expression and proviral DNA levels in U937 cells and in primary MDMs. The opioid antagonist naltrexone blocked the effect of fentanyl and reversed the expression of HIV protein and proviral DNA. Fentanyl led to a non-significant decrease in CXCR4 and CCR5 protein levels in U937 cells. RNA sequencing identified several differentially expressed genes in cells infected with HIV and exposed to fentanyl compared to infected cells with no drug exposure. Several microRNAs were also differentially expressed upon fentanyl exposure but not at a statistically significant level.

**Conclusion:**

These data demonstrate that the synthetic opioid fentanyl can promote HIV replication in macrophages. As higher HIV levels lead to accelerated disease progression and a higher risk of transmission to others, further research is needed to better understand opioid-virus interactions and to develop new and/or optimized treatment strategies for people living with HIV and opioid use.

## Introduction

In recent years, drug overdose deaths have increased significantly [1, 2]. The supply of synthetic opioids like fentanyl has increased the number of overdose deaths for more than a decade [3]. Since its commercial synthesis in 1960, fentanyl has become the most used opioid as an intraoperative analgesic [4]. Due to its high lipid solubility, fentanyl is more fast-acting and more potent than heroin, resulting in a quick onset of euphoria and relief from pain [5]. Nevertheless, fentanyl has become one of the most detected drugs involved in overdose fatalities in the United States [6]. Overdose deaths involving opioids accounted for 70.6% of deaths in 2019, while synthetic opioids accounted for 51.5%. According to the 2020 National Survey on Drug Use and Health, 9.5 million Americans misuse opioids every year, and 75% of the 92,000 drug overdose deaths were caused by opioids [7, 8].

Susceptibility to viruses such as HIV and viral hepatitis is common in people with opioid use disorders (OUDs), and several outbreaks have occurred as a result of the current opioid crisis [9, 10]. Multiple studies demonstrate that endogenous and exogenous opioids modulate immune function *in vitro* and *in vivo* [11–13] including multiple effects on adult macrophages [14]. Additionally, opioids such as morphine, methadone, buprenorphine, heroin, and fentanyl are thought to have immunomodulatory properties [15]. Several *in vitro* studies have demonstrated that opioids suppress multiple immune cells, including macrophage phagocytosis and cytokine secretion, neutrophil production of cytokines and chemokines, diminished cytotoxic function of natural killer cells, repression of dendritic cells, and decreased activation and proliferation of B and T cells [16, 17].

Macrophages and CD4^+^ T cells are major targets for HIV infection *in vivo* and play a major role in the pathogenesis of infection [18–20]. We have recently demonstrated that fentanyl increases the expression of HIV p24 antigen and enhances chemokine co-receptors in HIV-infected and HIV-susceptible lymphocytes [21]. However, the role of synthetic opioids such as fentanyl on HIV replication in macrophages is yet to be investigated.

## Methods

### Cell lines and reagents

U937 is a cell line exhibiting monocyte morphology that was obtained from ATCC (#CRL-1593.2). SH-SY5Y is a neuroblastoma cell line that robustly expresses the mu opioid receptor and was obtained from ATCC (#CRL-2266). Fentanyl and naltrexone were obtained as Certified Reference Material from Cerilliant (Round Rock, TX). Per their certificates of analysis, these reagents are suitable for the identification, calibration, and quantification of analytes in analytical and R&D applications. Fentanyl was diluted with dH_2_O to concentrations of 1 ng/mL, 100 ng/mL, and 10 ug/mL.

Human peripheral blood CD14^+^ monocytes were procured from Lonza and maintained in RPMI-1640 supplemented with heat-inactivated 10% fetal bovine serum (Biotechne, screened for MDM maturation and viability), 100 μg/mL streptomycin, 100 U/mL penicillin, 2 mM glutamine, and 5 ng/mL GM-CSF or 20 ng/mL M-CSF (Peprotech). Monocytes were maintained in cytokine-supplemented media for 7 days to facilitate maturation into monocyte-derived macrophages (MDMs).

### Mu opioid receptor expression

Mu opioid receptor expression (MOR) was quantified in 1 x 10^5^ cells using the human opioid receptor mu 1 (OPRM1) ELISA (MyBioSource; San Diego, CA) with a lower limit of detection (LOD) of 7.81 pg/mL.

### Virus preparation

HIV_YK-JRCSF_ (CCR5-tropic) was prepared by transfection of 1 x 10^6^ 293T cells (ATCC #CRL-3216) per well with 1 μg of the full-length HIV_YK-JRCSF_ plasmid [22] in a 6-well plate using the FuGene6 transfection reagent (Roche). Transfected cells were incubated at 37°C for an additional 48–72 hours. Virus-containing supernatants were passed through a 0.20 μm filter and precipitated in polyethylene glycol at 4°C. Virus was centrifuged at 14,000 g for 20 minutes, resuspended in phosphate-buffered saline (PBS), and frozen at -80°C until use. The virus was titered using TZM-bl cells and β -galactosidase staining. HIV p24 protein was quantified in culture supernatants as outlined below.

### HIV infection and p24 protein quantification

2 x 10^5^ U937 cells were infected with 0.5 TCID_50_ of HIV_YK-JRCSF_ for 1 hour. The virus was removed, and cells were washed with PBS 5 times to remove unbound virus prior to fentanyl exposure. HIV p24 protein was quantified using the HIV p24 ELISA Kit (Abcam; Cambridge, MA) with a lower limit of sensitivity of 1.1 pg/mL.

### Drug exposure

HIV-infected U937 cells were seeded at 2 x 10^5^ cells per well. Fentanyl was added to the culture medium after 24 hours. After 24 hours of incubation with drug, p24 protein was quantified in culture supernatants by ELISA.

### Proviral DNA quantification

DNA was extracted from ACH-2 cells that contain a single copy of HIV proviral DNA (LAV strain) and used as a positive control to quantify virus levels in U937 cells before and after fentanyl exposure. HIV proviral DNA copy number was quantified by real-time PCR amplification using Brilliant III ultra-fast SYBR green QPCR master mix (Agilent) as described elsewhere [23]. Primer sequences used for amplification of HIV-1 pol: 5′ - TAC AGG AGC AGA TGA TAC AG - 3′ and 5′ - CCT GGC TTT AAT TTT ACT GG - 3′. SYBR green real-time PCR assay was performed in 20 μL of PCR mixture consisting of 2x Master Mix, 200 nM of each oligonucleotide primer targeting the HIV pol gene, and DNA extracted from U937 cells treated and untreated with HIV _YK-JRCSF_ +/- fentanyl at three different concentration. To quantify HIV provirus, a standard curve was defined using serial dilutions of ACH-2-derived DNA ranging from 1 to 10^6^ copies/uL. All standard dilutions, controls, and samples were run in triplicate, and the average value ct was utilized to quantify HIV DNA.

Human peripheral blood CD14^+^ monocytes were obtained from Lonza and cultured in RPMI 1640. Cell counting was performed using trypan blue staining and 1 x 10^6^ cells were seeded in a flask and supplemented with RPMI 1640 + GM CSF+ IL-4 and incubated at 37°C for 6 days for monocyte to macrophage transformation. On day 7, MDMs were harvested, and 1 x 10^5^ cells per well were plated.

Naltrexone was added (10 μg/mL) and incubated for 1–2 hours and then fentanyl (10 μg/mL) was added and incubated for 24 hours. After 24 hours, cells were infected with HIV_YK-JRCSF_ and incubated for 2 hours. Cells were rinsed three times to remove any unbound virus and replaced with fresh media with fentanyl and incubated for 3 days. Supernatant and cells were harvested at the end of 72 hours. HIV p24 ELISA was performed with supernatants, and PCR for detection of integrated DNA was performed from DNA extracted from cell lysates by real-time PCR based on SYBR Green I detection.

### Chemokine receptor expression

CCR5 protein expression was quantified in cell lysates using the Human CC-Chemokine Receptor 5 (CCR5) ELISA Kit (MyBioSource.com; catalog #MBS166945) with a sensitivity of 2.61 ng/L. CXCR4 protein expression was quantified in cell lysates using the Human CXC-Chemokine Receptor 4 (CXCR4) ELISA Kit (MyBioSource.com; catalog #MBS771091) with a sensitivity of 3.9 pg/mL.

### TLR9 receptor expression

TLR9 protein expression was quantified in cell lysates by the Human Toll-Like Receptor 9 ELISA Kit (MyBioSource.com; catalog #MBS762185) with a sensitivity of 0.094 ng/mL.

### Cell viability

10,000 U937 cells were seeded per well of a 96-well plate. Fentanyl was added to the culture medium after 24 hours. After 24 hours of incubation with fentanyl, the potential toxicity was evaluated by the MTT Cell Proliferation Assay Kit (BioVision; Milpitas, CA; catalog #K299-1000).

### RNAseq and microRNAseq

The U937 cell line was seeded at 1 x 10^6^ cells per well. 100 ng/mL fentanyl was added to the culture medium after 24 hours. After 24 hours of incubation with drug, total RNA was isolated using the commercially available microRNA Isolation Kit (miVana; Applied Biosystems; Carlsbad, CA) according to the manufacturer’s protocol. Total RNA concentration and purity were determined by NanoDrop 2000 spectrophotometer (Thermo Fisher Scientific; Waltham, MA). A 2100 Bioanalyzer (Agilent; Santa Clara, CA) and agarose electrophoresis were used to assess RNA integrity.

MicroRNAseq was performed by the Genomics, Epigenomics and Sequencing Core at the University of Cincinnati [24]. NEBNext small RNA sample library preparation kit (NEB; Ipswich, MA) was used to prepare the library with a modified approach for precise microRNA library size selection and higher library recovery and microRNA read alignment. After 15 cycles of PCR with 100 ng total RNA as input, the libraries with unique indices were pooled, column cleaned, and combined with a custom-designed DNA ladder containing purified amplicons of 135 and 146 base pairs (bp). This size range corresponds to a 16–27 nt insert microRNA library that covers all microRNAs. The library pool ranging from 135 to 146 bp, including the DNA marker, was gel purified and quantified using the NEBNext Library Quant kit (NEB) in the QuantStudio 5 Real-Time PCR System (Thermo Fisher Scientific; Waltham, MA). A few million reads were generated to determine the relative concentration of each library using an Illumina Nextseq 550 sequencer. To achieve equal numbers of reads from each sample, the capacity of each library was adjusted for the final data analysis. Sequences were pre-processed using the ShortRead R tool to remove adapters and low-quality reads. Using the Bowtie2 aligner, reads were aligned to the reference human genome(hg19) [25]. The reads aligning to each known mature microRNA were counted using Bioconductor packages for next-generation sequencing data analysis [26] based on microRNA definitions in the miRBase database [27]. Statistical analysis to detect differentially expressed microRNAs were performed, and p-values were calculated based on the negative binomial model of read counts as implemented in edgeR [28].

Following established protocols, the University of Cincinnati’s Genomics, Epigenomics and Sequencing Core carried out directed polyA RNA sequencing [29, 30]. Bioanalyzer was used to test the quality of total RNA (Agilent; Santa Clara, CA). The Poly(A) mRNA Magnetic Isolation Module (NEBNext; Ipswich, MA) was used to isolate polyA RNA for library preparation with 1 μg of high-quality total RNA as input. SMARTer Apollo automated NGS library prep system (Takara Bio USA; Mountain View, CA) and the New England BioLabs NEBNext Ultra II Directional RNA Library Prep kit (PCR cycle number 8) were utilized for enrichment of polyA RNA and library preparation, respectively. Following QC and real-time qPCR (NEBNext Library Quant Kit; New England BioLabs; Ipswich, MA), the individual indexed libraries were proportionally pooled and sequenced using an Illumina NextSeq 550 sequencer (Illumina; San Diego, CA) at single read 1x85 bp setting. Using Kallisto the raw reads were aligned, which uses pseudoalignment to determine if they are compatible with genomic targets. Genomic annotations were provided by the University of California, Santa Cruz (UCSC) Genome Browser, with output in transcripts per million (TPM). Raw data were log_2_-transformed and baselined to the median of all samples. Transcripts were filtered further to include only those with TPM > 3 in 50% of samples (N = 10,703 transcripts). The level of differential expression was determined using moderated t-tests [31] with a significance cutoff of p < 0.05. With a focus on biological processes and pathways, significant transcripts were evaluated for their ontological relevance using ToppGene and ToppCluster, and figures were produced in Cytoscape. To construct comprehensive gene sets for candidate biological functions, Gataca (https://gataca.cchmc.org/gataca/) and ToppGene were used (https://toppgene.cchmc.org), where the input for Gataca is a biological process or pathway and the output is related genes. Principal component analysis (PCA) was then used to create principal components from candidate gene sets, which were plotted to assess each candidate gene set’s capacity to separate samples based on drug exposure.

### Quantitative real time PCR validation

SYBR green analysis was utilized to validate the accuracy of the RNAseq results using quantitative real-time PCR (qRT-PCR). Total cellular RNA was extracted from cells infected with HIV and treated with fentanyl / no fentanyl with RNeasy Mini Kit (Qiagen, Valencia, CA) according to the manufacturer’s instructions. DNA synthesis and PCR amplification were performed with the Brilliant III Ultra-Fast SYBR Green qPCR Master Mix (#600887, Agilent Technologies, Santa Clara, CA, USA). Beta globin was used as the endogenous control for mRNA analysis. Two upregulated (*AGAP5*, *EIF3C*) and two downregulated genes (*EARS2*, *DDX42)* were selected. The primers used are listed in S1 Table. Amplifications were performed with the following thermal cycling conditions: 10-minute incubation at 50°C for cDNA synthesis followed by initial activation at 95°C for 10 minutes, 40 cycles of 30 seconds (sec) at 95°C, 30 seconds of Tm, and 1 minute at 60°C. A dissociation curve was generated to confirm the generation of a single PCR product. Gene expression was calculated using the 2−ΔΔct method and normalized to a housekeeping gene.

### Statistical analysis

For each experimental condition, technical duplicates were performed with error bars representing the standard deviation. ANOVA with replication was utilized to evaluate the statistical significance (p < 0.05) for the different doses of fentanyl compared to the “no drug” condition.

## Results

### Expression of the mu opioid receptor

**Fig 1:**
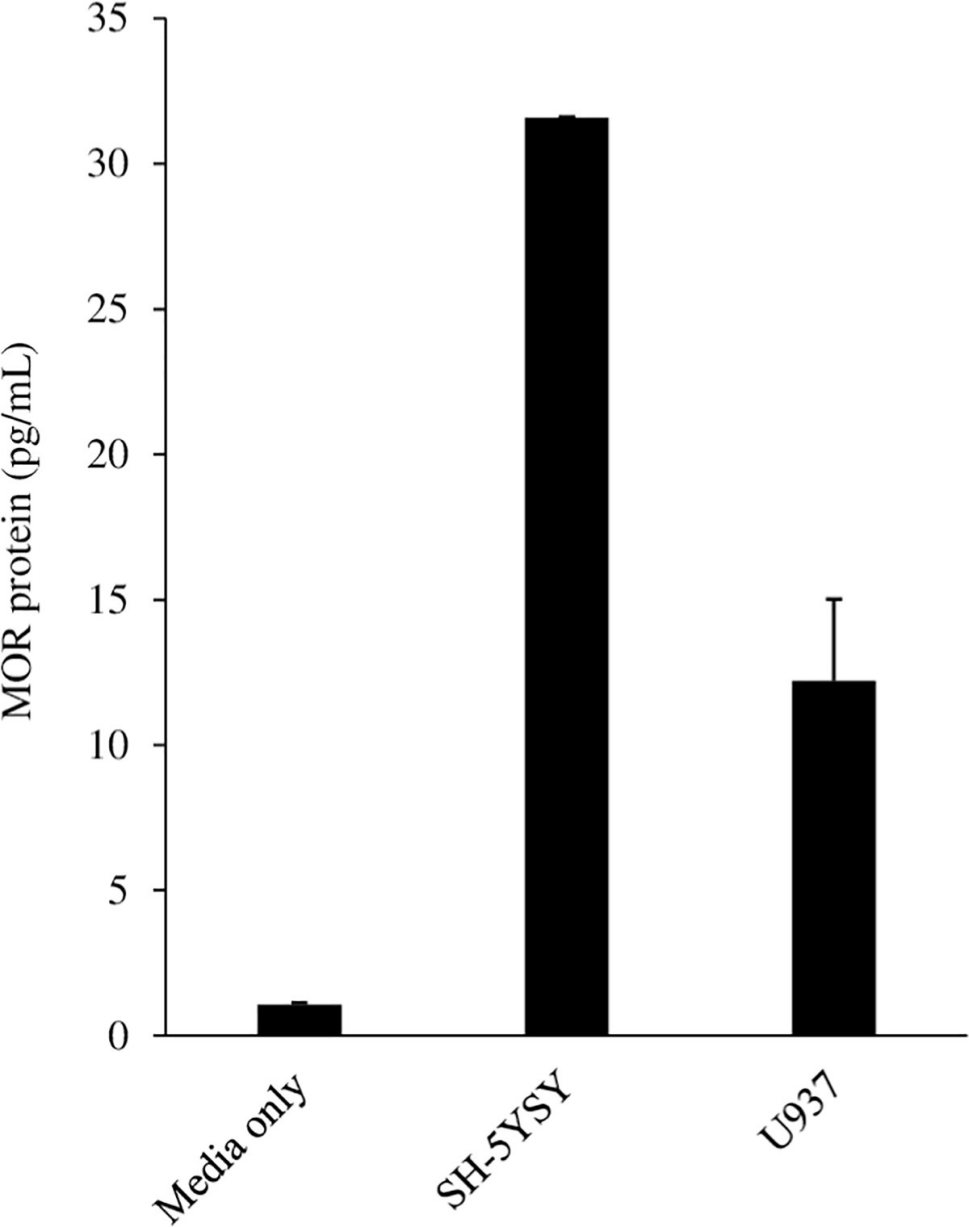
Mu opioid receptor (MOR) expression (pg/mL) was quantified by ELISA in 1 x 10^5^ cells resuspended in 100 μL lysis buffer and diluted 1:2.

Mu opioid receptor (MOR) expression was quantified in U937 cells. MOR expression was lower than in the positive control SH-SY5Y neuroblastoma cell line but still robustly expressed (Fig 1).

### Fentanyl enhances HIV replication

**Fig 2:**
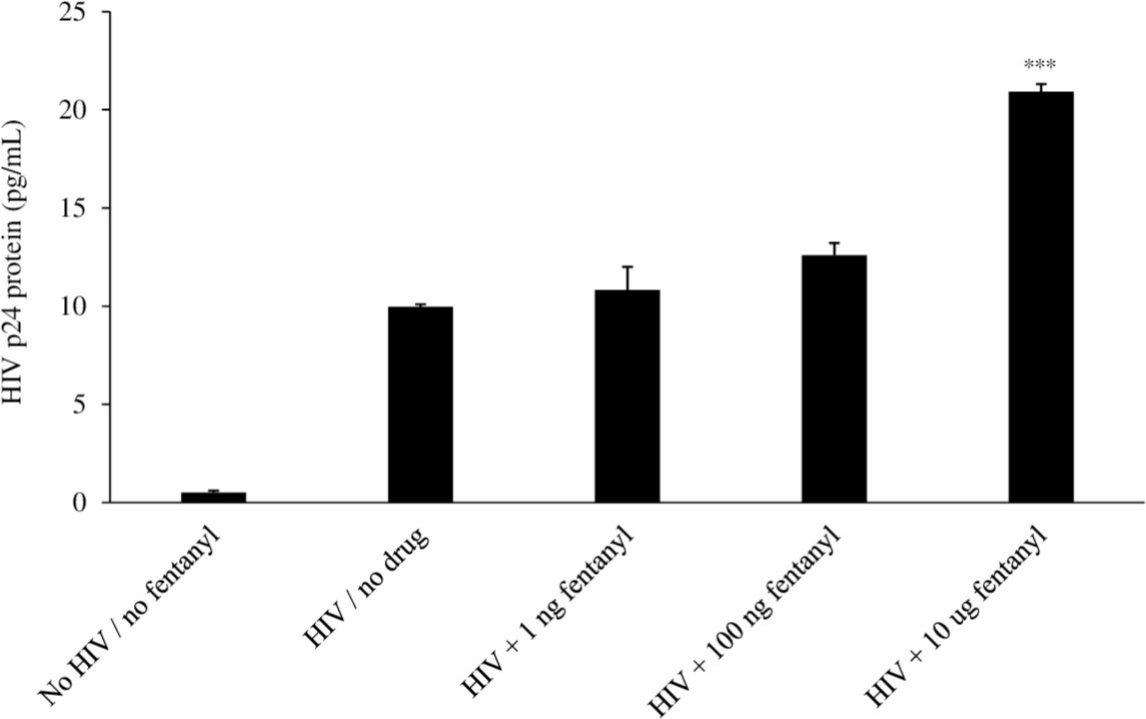
The U937 cell line was seeded at 1 x 10^5^ cells per well. Cells were infected with HIV_YK-JRCSF_ at a TCID_50_ of 0.5 for 1 hour and rinsed with PBS three times to remove any unbound virus and replaced with DMEM. 0 ng, 1 ng, 100 ng, or 10 μg of fentanyl was added to the culture medium after 24 hours. After 24 hours of incubation with drug, HIV p24 protein (pg/mL) was quantified in culture supernatants. ANOVA for dose effect = 0.0038.

**Fig 3:**
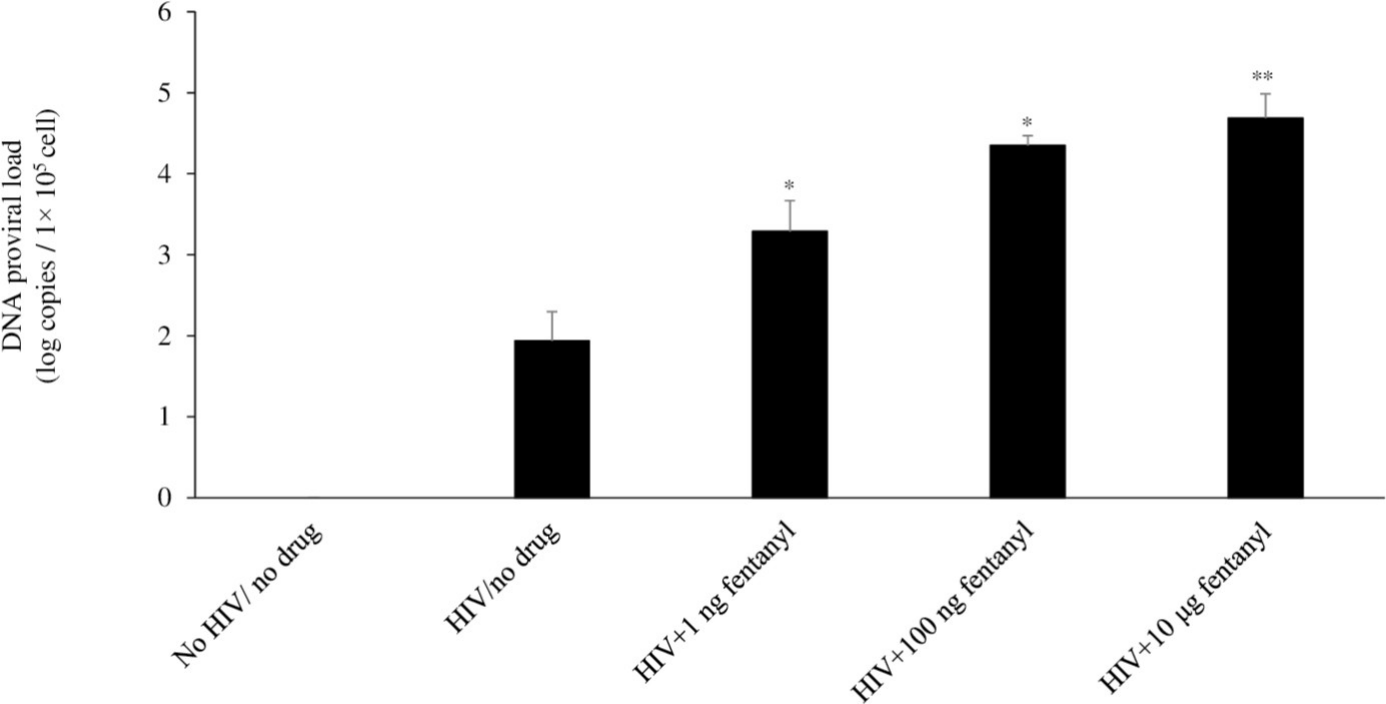
U937 cells were seeded at ~1 × 10^5^ cells per well. After 24 hours, cells were treated with HIV_YK-JRCSF_ at TCID_50_ of 0.5 for 1 hour and rinsed with PBS three times to remove any unbound virus and replaced with fresh DMEM. 0 ng, 1 ng, 100 ng, or 10 μg of fentanyl was added to the culture medium after 24 hours. After incubation with drug for 24 hours, HIV proviral DNA was quantified in cells by real-time PCR based on SYBR Green I detection. Error bars represent the standard deviations between replicates. ANOVA for dose effect = 0.0001.

Previous studies have shown that other drugs of abuse increase long terminal repeat activation and/or HIV gene expression in monocytes/macrophages [32–34]. Thus, we evaluated the potential impact of fentanyl on HIV expression. As shown in Fig 2, fentanyl led to a dose-dependent increase in HIV p24 protein expression at 24 hours post-drug exposure (ANOVA for dose effect = 0.0038). Log_10_ copies of HIV proviral DNA were also increased with dose-dependent concentration of drug compared to HIV-infected but drug-naïve U937 cells (ANOVA for dose effect = 0.0001) (Fig 3).

### Fentanyl alters expression of HIV co-receptors

**Fig 4:**
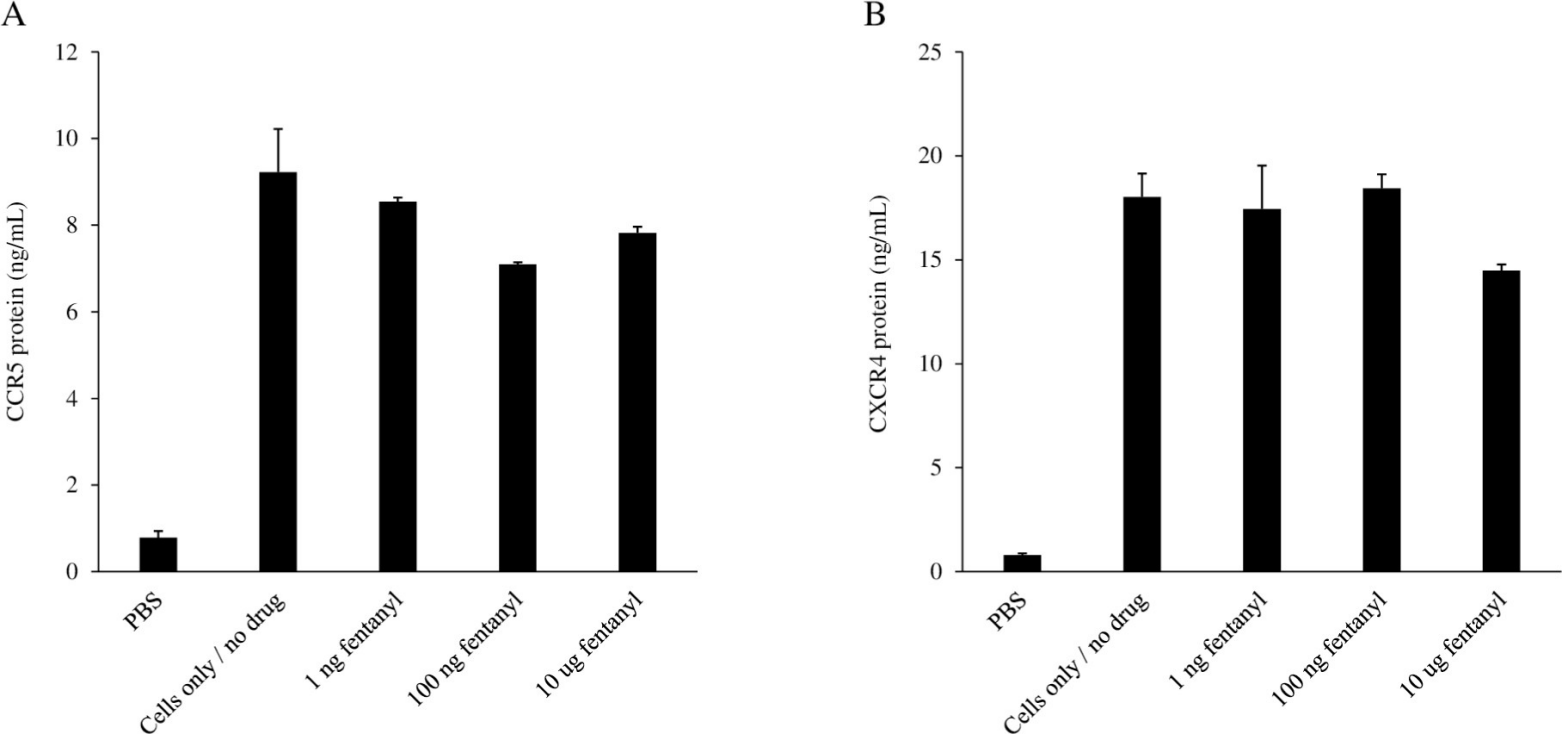
The U937 cell line was seeded at 2 x 10^5^ cells per well. 0 ng, 1 ng, 100 ng, or 10 μg of fentanyl was added to the culture medium after 24 hours. After 24 hours of incubation with drug, **(A)** CCR5 receptor (ng/mL) and **(B)** CCR4 receptor protein (ng/mL) levels were quantified by ELISA in cell lysates. ANOVA for dose effect = 0.0946 for CCR5 and 0.1414 for CXCR4.

As the interaction of HIV with susceptible cells is mediated by its primary receptor (CD4) and chemokine co-receptors (CCR5 and CXCR4), we investigated the influence of fentanyl on CCR5 and CXCR4 protein expression levels in HIV-uninfected U937 cells treated with drug. We previously reported increased expression of CCR5 and CXCR4 in other cell types [35]; however, in U937 cells, fentanyl resulted in a modest but non-significant decrease in CCR5 protein expression (Fig 4A; ANOVA for dose effect = 0.0946). There was no change in CXCR4 protein expression in U937 cells treated with fentanyl (Fig 4B; ANOVA for dose effect = 0.1414).

### Fentanyl does not impact TLR9 expression

**Fig 5:**
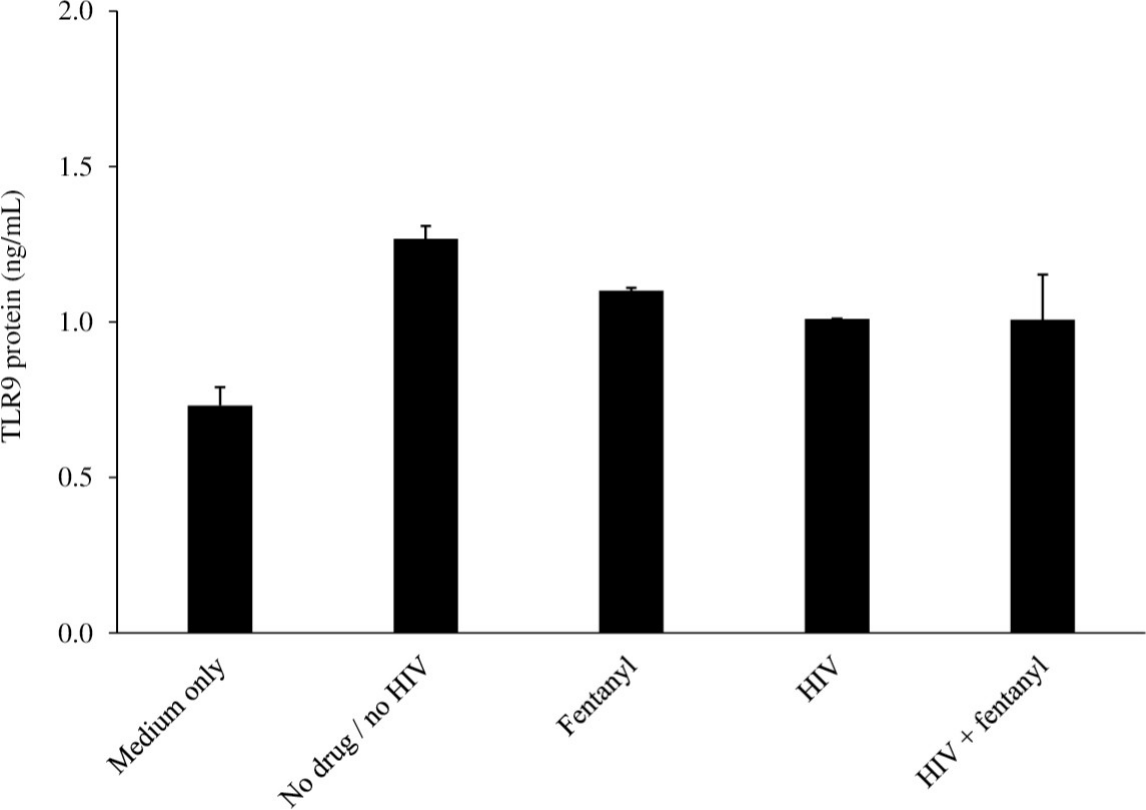
The U937 cell line was seeded at 2 x 10^5^ cells per well. 100 ng fentanyl was added to the culture medium after 24 hours. After 24 hours of incubation with drug, cells were infected with HIV_YK-JRCSF_ at a TCID_50_ of 0.5 for 1 hour and rinsed with PBS three times to remove any unbound virus and replaced with fresh DMEM. After 24 hours of incubation, TLR9 receptor protein (ng/mL) was quantified by ELISA in cell lysates. ANOVA for dose effect = 0.455.

Liao *et al*. previously reported that opioid use leads to decreased expression of toll-like receptor 9 (TLR9) in individuals with HIV and that morphine promoted HIV replication in macrophages by inhibiting TLR9 [36]. We previously observed that HIV infection of SH-SY5Y cells resulted in a significant increase in TLR9 protein expression and that this induction was abolished by fentanyl [37]. In contrast, no such effect of fentanyl on TLR9 expression was observed in U937 cells (Fig 5; ANOVA for dose effect = 0.455).

### Fentanyl decreases cell viability

**Fig 6:**
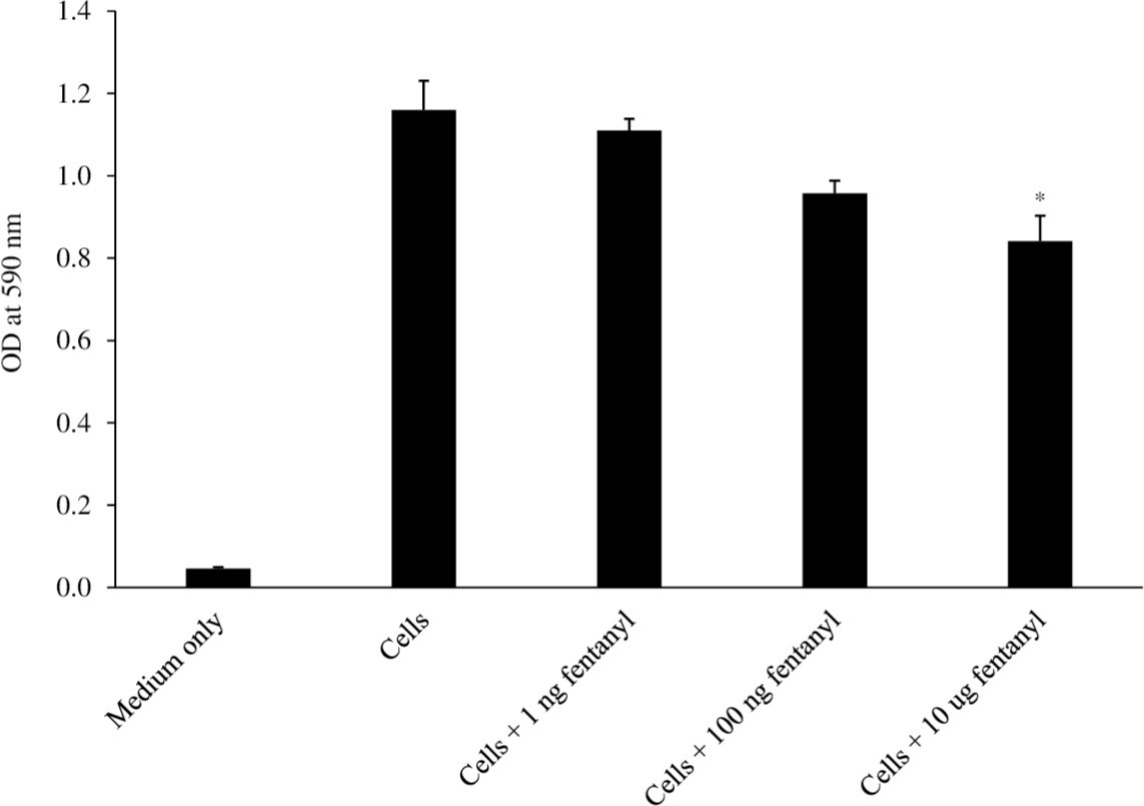
The U937 cell line was seeded at 1 x 10^4^ cells per well. Fentanyl was added to the culture medium after 24 hours. After 24 hours of incubation with drug, the potential toxicity (optical density [OD] at 590 nm) was evaluated by the MTT Cell Proliferation Assay Kit. ANOVA for dose effect = 0.0402.

Given that fentanyl could impact cell growth, cell proliferation was quantified in U937 cells exposed to fentanyl. As shown in Fig 6, a modest dose-dependent reduction in U937 cell number was observed, although cell viability remained high (ANOVA for dose effect = 0.0402).

### Fentanyl had no significant effect on microRNA expression

Using total microRNA isolated from U937 cells treated with or without fentanyl for 24 hours, a comprehensive microRNA expression profile was generated. There were 19 microRNAs differentially expressed with p-value < 0.2 as shown in S1 Fig. A list of microRNAs with their p-value and fold change is provided in S2 Table.

### Fentanyl alters the cellular transcriptome in U937 cells

**Fig 7:**
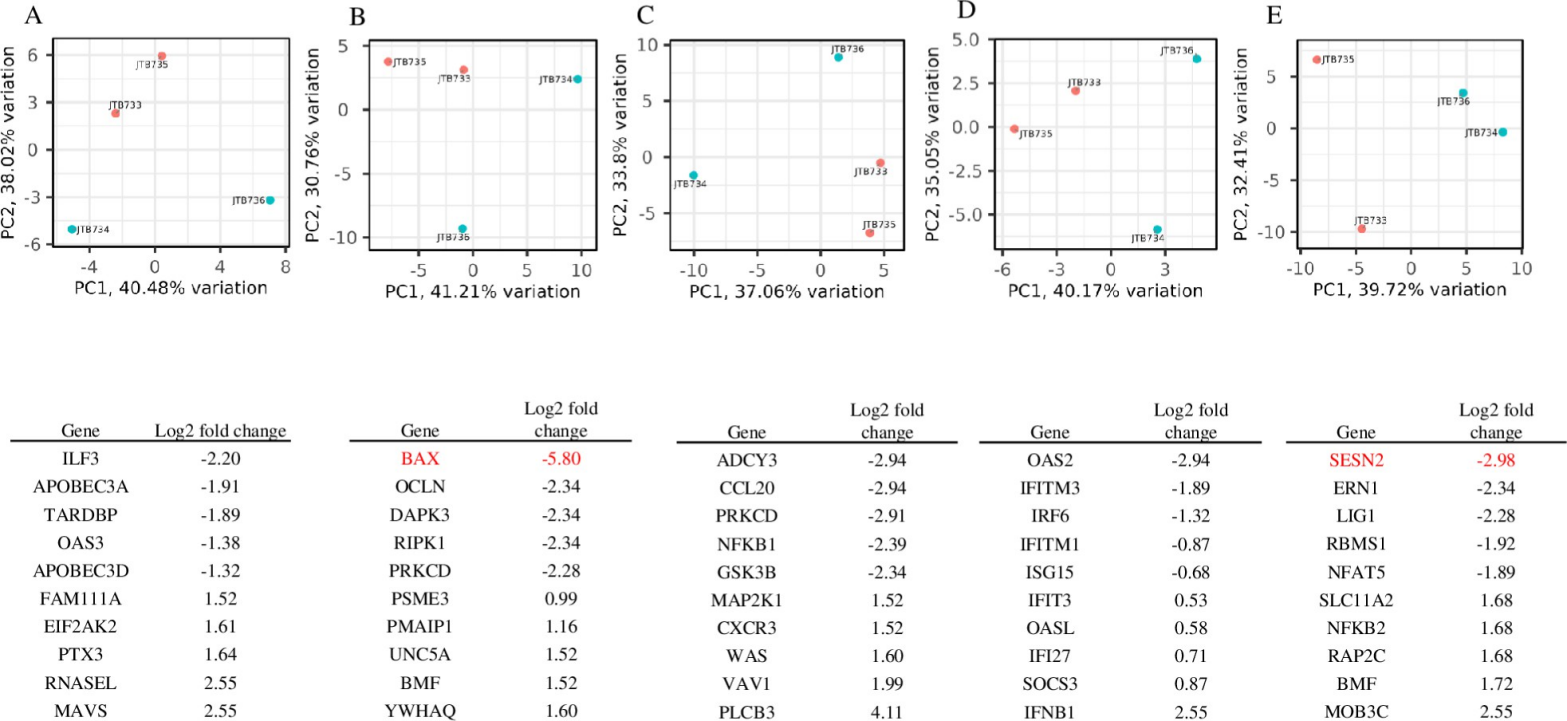
Principle component analysis of the expression of mRNA in U937 cells in the presence / absence of fentanyl. **(A)** Antiviral, **(B)** cell death, **(C)** chemokine, **(D)** interferon, and **(E)** NFκB signaling genes that are significantly differentially expressed (red circle = no fentanyl and cyan circle = fentanyl treated). Genes highlighted in red are those that are statistically significant.

To further explore the role of fentanyl in HIV pathogenesis, U937 cells were treated with fentanyl to characterize its effect on cellular gene expression. Differential analysis identified 14,740 transcripts of which 56 were significantly expressed in the fentanyl-treated cells compared to untreated. The list of transcripts that are significantly upregulated or downregulated is shown in S3 Table. GO enrichment for the differentially expressed genes using ToppGene is provided in S4 Table. Principal component analysis (PCA) was plotted to determine each candidate gene set’s ability to segregate samples according to drug exposure (Fig 7). Using principal component analysis, gene expression profiles were then explored in more depth for genes involved in antiviral response, cell death, chemokine signaling, interferon response, and NFκB signaling. The list of genes under each subcategory is provided in S5 Table. A list of five upregulated genes with the highest fold change and downregulated genes with the lowest fold change is provided in Fig 7.

### Validation of RNAseq data by real-time PCR

To validate the RNAseq results, two sets of differentially expressed genes that demonstrated up regulation or down regulation, coupled with a low P value (P value < 0.05) were selected for further analysis. The analysis of real-time PCR results using RNA isolated from both U937 and MDM cells infected with HIV and treated with fentanyl revealed overall agreement with the RNAseq data. Variations in the expression pattern of the differentially expressed genes were observed in the real-time PCR analysis among the RNA samples isolated from U937 and MDM cells. Expression of *AGAP4*, *EIF3C*, *DDX42*, and *EARS2* genes were statistically significant in U937 cells (S2 Fig). Except for the *EIF3* gene, all other three genes showed significant expression in MDMs (S3 Fig).

### Fentanyl enhances HIV replication in monocyte-derived macrophages

**Fig 8:**
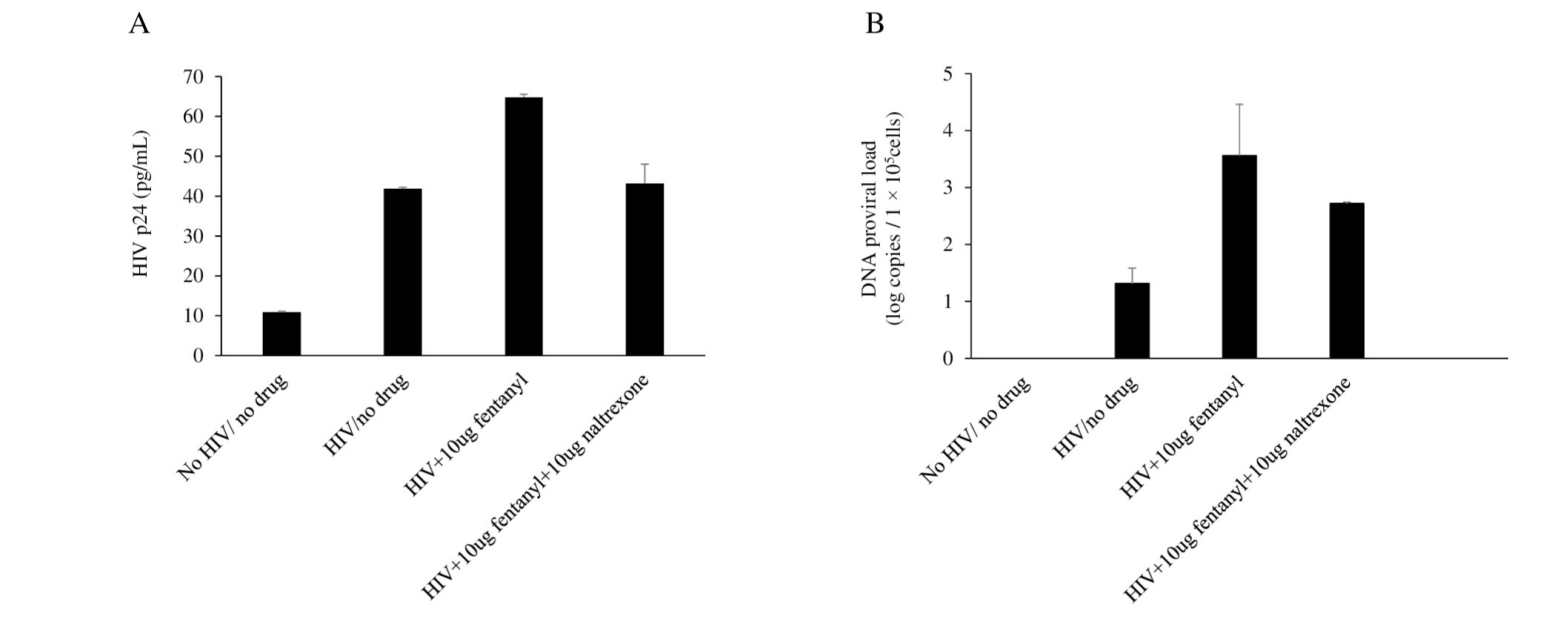
Primary monocytes at 1 x 10^6^ cells were seeded in a flask and supplemented with RPMI 1640 + GM-CSF + IL-4 and incubated at 37°C for 6 days for monocyte to macrophage transformation. On day 7, monocyte-derived macrophages [MDM] were harvested, and 1 x 10^5^ cells per well were plated. Naltrexone was added at 10 ug/mL and incubated for 1–2 hours, and then fentanyl at 10 ug /mL was added and incubated for 24 hours. After 24 hours, cells were infected with HIV_YK-JRCSF_ and incubated for 2 hours. The cells were then rinsed three times to remove any unbound virus and replaced with fresh media with fentanyl and incubated for 3 days. Supernatant and cells were harvested at the end of 72 hours. **(A)** HIV p24 ELISA was performed from supernatant, ANOVA for dose effect = 0.0066 and **(B)** integrated DNA was quantified from cells by real-time PCR based on SYBR Green I detection, ANOVA for dose effect = 0.0558. Error bars represent the standard deviations between replicates.

We further tested the effects of fentanyl on HIV-infected MDMs (Fig 8). Fentanyl treatment resulted in a 54.8% increased expression of HIV p24 and 2.25 log_10_ increase in proviral DNA. Naltrexone is an opioid antagonist used to treat opioid and alcohol use disorder and can limit HIV replication induced by opioids [38]. Treatment with 10 ug/mL naltrexone blocked the effect of fentanyl on HIV replication.

## Discussion

In recent years, there has been a significant increase in deaths related to drug overdoses. Numerous studies have shown that opioids depress immunity and increase infection susceptibility [39]. Opioid use disorders (OUDs) increase the risk of contracting infections such as HIV and viral hepatitis [11]. The immune function can be modulated by endogenous opioids and exogenous opioids [11]. For instance, HIV replication was elevated in morphine-exposed immune cells [14, 40, 41]. Morphine may affect HIV disease by modulating beta-chemokine, chemokine receptor, and microRNA expression mechanisms [42, 43]. Cocaine has also been reported to have similar effects on HIV replication [44]. Cocaine activates HIV and promotes replication by downregulation of chemokines [45]. Similarly, HIV replication is facilitated by methamphetamine [46, 47]. Fentanyl users are likely to inject and share syringes frequently, which has become a major concern for disease prevention efforts since this increases the risk of contracting parenterally transmitted diseases such as HIV and hepatitis C [48–50]. Macrophages and CD4^+^ T cells are major targets for HIV and play a major role in pathogenesis. We have recently demonstrated that fentanyl increases HIV p24 antigen production and enhances chemokine co-receptors in HIV-infected and HIV-susceptible CD4^+^ T cells [21]. To explore the effect of fentanyl on the other immunological cell types involved in HIV infection pathogenesis, we evaluated macrophages.

A previous study by Wang X *et al*. performed in peripheral blood mononuclear cells demonstrated that morphine activates the HIV long terminal repeat (LTR) and reduces anti-HIV microRNA levels [51]. Additionally, cocaine can enhance HIV transcription, raising virus load in patients [52, 53]. Morphine and methadone promote HIV replication in human immune cells by stimulating the expression of CCR5, a principal HIV entry coreceptor. Earlier research has shown that morphine promotes HIV infection by upregulating CCR5 expression in macrophages [54]. Methadone can also increase CCR5 production and enhance HIV infection [33, 55]. Thus, fentanyl may enhance HIV entry into cells by increasing HIV co-receptor expression. While we previously reported increased expression of CCR5 and CXCR4 in other cell types [21, 37], in the current study fentanyl resulted in a modest but non-significant decrease in CCR5 protein expression in U937 cells and there was no major difference in CXCR4 protein expression.

Naltrexone is a MOR antagonist approved by the FDA for alcohol use disorder and OUD [56]. In persons living with HIV, naltrexone extends viral suppression of HIV [57]. *In vitro*, naltrexone reverses enhanced viral replication induced by several drugs of abuse [58]. Supporting these findings, our results also demonstrated that both HIV p24 protein and proviral DNA expression decreased when opioid receptors were blocked with naltrexone.

Our previous studies demonstrated that fentanyl inhibited HIV-induced TLR9 expression [37]. Several TLRs may be involved in the pathogenesis of HIV [59]. Earlier reports showed that the clinical course of HIV was shown to be impacted by TLR9, although further study is required to determine how TLR9 influences HIV [60]. A study by Brichacek *et al*. found that TLR9 agonists reduced HIV replication in PBMCs [61]. A study by Liao *et al*. found that opioids reduced the expression of TLR9 within HIV-infected individuals [36]. Methamphetamine also interferes with TLR9-mediated HIV antiviral activity [62]. As a result of downregulating TLR expression in host cells, fentanyl suppresses HIV immunity. However, our study did not show any effect of fentanyl on TLR9 expression in U937 cells.

Limitations of the present study should be considered. First, *in vitro* exposure to fentanyl was brief (24 hours) and may not reflect chronic, long-term drug use. There is also a high rate of polysubstance use among persons who inject drugs. Thus, fentanyl use may occur alongside other substances such as methamphetamine and cocaine. Second, despite a lack of well-characterized physiologically relevant concentrations of fentanyl, other researchers have reported concentrations between 10^−12^ M and 10^−4^ M [63–65]. We used three different concentrations of fentanyl in this experiment, which is in line with the values found in patient populations with drugs of abuse. Third, our experiments used a single HIV isolate; however, it is possible that distinct viral isolates may respond differently to fentanyl. Fourth, there are many metabolites and analogs of fentanyl that should be examined as well. In addition to their distinctive binding affinities for mu opioid receptors, analogs and metabolites may also inhibit HIV replication [66, 67]. Finally, RNA sequencing results showed multiple transcripts that were differently expressed in the cells treated with fentanyl compared to untreated HIV-infected U937 cells. Several microRNAs were also differentially expressed, although none were significant.

Taken together, our study provides compelling experimental evidence that fentanyl enhances HIV infection and replication in U937 and primary MDM cells. Further, the effect of fentanyl was found to be reversed by naltrexone. This proved that the deleterious function of fentanyl on HIV pathogenesis could be altered by blocking the MOR on the target cells. Comprehensive research is required to understand the molecular mechanisms underlying fentanyl’s promotion of HIV replication and infection *in vivo*.

## Supporting information

S1 Table List of primers used to validate RNA sequencing data. (RTF)

S2 Table MicroRNAs with p value <0.2 in U937 cells infected with HIV in the presence / absence of fentanyl. (DOCX)

S3 Table Transcripts that are significantly upregulated or downregulated in U937 cells infected with HIV in the presence / absence of fentanyl. (DOCX)

S4 Table GO enrichment for the differentially expressed genes using ToppGene in U937 cells infected with HIV in the presence / absence of fentanyl. (RTF)

S5 Table Differentially expressed genes in U937 cells infected with HIV in the presence / absence of fentanyl by gene function (antiviral response, cell death, chemokine signaling, interferon response, and NFκB signaling). (XLSX)

S1 Fig **(A)** Volcano plot of all microRNAs in U937 cells in the presence / absence of fentanyl. **(B)** Heatmap of microRNAs that are differentially expressed in U937 cells in the presence / absence of fentanyl (p value < 0.2). Those microRNAs that are upregulated are shown in red, while those that are downregulated are shown in green.
(PDF)

S2 Fig Primary monocytes at 1 x 10^5^ cells were seeded in a flask and supplemented with RPMI 1640 + GM-CSF + IL-4 and incubated at 37°C for 6 days for monocyte to macrophage transformation. On day 7, monocyte-derived macrophages [MDM] were harvested, and 1 x 10^5^ cells per well were plated. Fentanyl at 10 ug/mL was added and incubated for 24 hours. After 24 hours, cells were infected with HIV_YK-JRCSF_ and incubated for 2 hours. The cells were rinsed three times to remove any unbound virus and replaced with fresh media with fentanyl and incubated for 3 days. Cells were harvested at the end of 72 hours and total RNA extraction was performed. Genes of interest were quantified by brilliant III ultrafast SYBR qRT-PCR. Error bars represent the standard deviations between replicates. Data were normalized to beta-globin expression and fold-change in expression was calculated by the 2 ^−ΔΔCT^ method. *p < 0.05; **p < 0.01; ***p < 0.001; ****p < 0.0001.
(PDF)

S3 Fig U937 at 1 x 10^5^ cells per well were plated. Fentanyl at 10 ug/mL was added and incubated for 24 hours. After 24 hours, cells were infected with HIV_YK-JRCSF_ and incubated for 2 hours. The cells were rinsed three times to remove any unbound virus and replaced with fresh media with fentanyl and incubated for 3 days. Cells were harvested at the end of 72 hours and total RNA extraction was performed. Genes of interest were quantified by brilliant III ultrafast SYBR qRT-PCR. Error bars represent the standard deviations between replicates. Data were normalized to beta-globin expression and fold-change in expression was calculated by the 2 ^−ΔΔCT^ method. *p < 0.05; **p < 0.01; ***p < 0.001; ****p < 0.0001.
(PDF)
