# Supplementary material for: The synthetic opioid fentanyl increases HIV replication in macrophages
Source: PLoS One. 2025 Feb 27;20(2):e0298341. doi: 10.1371/journal.pone.0298341 (PMC11867328; doi:10.1371/journal.pone.0298341)
Supplement: S1 Table — (RTF) [file pone.0298341.s001.rtf]

S1 table. List of primers used to validate RNA sequencing data.
Gene	Forward Sequence	Reverse Sequence	
DDX42	GGAGGGTCATAATCTTGGGCTG	TGAAGGAATGTCCAGACCACGG	
EIF3c	GGAAGTGCCTGGACTGCATCAA	CCATTCGTTCCACCAGAGTTAGG	
EARS2	AGGTGGATGTGATTGCCAAGCG	GTACTTGGTGCCTTCCAGACCT	
AGAP5	CCACATTCGTGACCAGGAGATG	TTGTGCTTGCCTCTGGATTGGC	
