# Supplementary material for: The synthetic opioid fentanyl increases HIV replication in macrophages
Source: PLoS One. 2025 Feb 27;20(2):e0298341. doi: 10.1371/journal.pone.0298341 (PMC11867328; doi:10.1371/journal.pone.0298341)
Supplement: S2 Table — (DOCX) [file pone.0298341.s002.docx]

**S2 table.** MicroRNAs with p value <0.2 in U937 cells infected with HIV in the presence / absence of fentanyl.

| Gene ID | MicroRNA name | Log_2_ fold change | P value |
| --- | --- | --- | --- |
| MIMAT0014987 | hsa-miR-548s | -1.48 | 0.08 |
| MIMAT0022490 | hsa-miR-5697 | 2.65 | 0.10 |
| MIMAT0027632 | hsa-miR-6866-5p | -1.48 | 0.11 |
| MIMAT0011159 | hsa-miR-2115*; hsa-miR-2115-3p | 2.01 | 0.11 |
| MIMAT0004808 | hsa-miR-625*; hsa-miR-625-3p | -1.38 | 0.13 |
| MIMAT0015028 | hsa-miR-3154 | -1.41 | 0.13 |
| MIMAT0004955 | hsa-miR-374b; hsa-miR-374b-5p | 1.02 | 0.13 |
| MIMAT0022735 | hsa-miR-374c-3p | 1.02 | 0.14 |
| MIMAT0015031 | hsa-miR-3157; hsa-miR-3157-5p | 1.86 | 0.14 |
| MIMAT0000243 | hsa-miR-148a; hsa-miR-148a-3p | -0.93 | 0.15 |
| MIMAT0003293 | hsa-miR-624; hsa-miR-624*; hsa-miR-624-5p | 1.49 | 0.16 |
| MIMAT0003327 | hsa-miR-449b; hsa-miR-449b-5p | 1.85 | 0.16 |
| MIMAT0004982 | hsa-miR-939; hsa-miR-939-5p | 1.66 | 0.18 |
| MIMAT0000089 | hsa-miR-31; hsa-miR-31-5p | 1.17 | 0.19 |
| MIMAT0000681 | hsa-miR-29c; hsa-miR-29c-3p | 1.19 | 0.19 |
| MIMAT0000444 | hsa-miR-126*; hsa-miR-126-5p | 1.55 | 0.19 |
| MIMAT0021083 | hsa-miR-5091 | -1.26 | 0.19 |
| MIMAT0021086 | hsa-miR-5094 | 1.66 | 0.19 |
| MIMAT0027367 | hsa-miR-6733-5p | -1.17 | 0.20 |
