# Supplementary material for: The synthetic opioid fentanyl increases HIV replication in macrophages
Source: PLoS One. 2025 Feb 27;20(2):e0298341. doi: 10.1371/journal.pone.0298341 (PMC11867328; doi:10.1371/journal.pone.0298341)
Supplement: S3 Table — (DOCX) [file pone.0298341.s003.docx]

**S3 table.** Transcripts that are significantly upregulated or downregulated in U937 cells infected with HIV in the presence / absence of fentanyl.

| gene | baseMean | log2FoldChange | pvalue |
| --- | --- | --- | --- |
| *EARS2* | 63.02 | -1.196252729 | 0.001 |
| *LENG8* | 649.32 | -0.249692989 | 0.002 |
| *PPIA* | 2756.77 | -0.212059355 | 0.000 |
| *RPL12* | 2803.20 | -0.000126215 | 0.035 |
| *TMSB4X* | 4331.42 | -3.71E-05 | 0.022 |
| *RPS17* | 5253.88 | -3.64E-05 | 0.033 |
| *GOLGA8N* | 1298.08 | -3.58E-05 | 0.009 |
| *RPL22* | 1830.94 | -3.40E-05 | 0.008 |
| *CALR* | 3176.29 | -2.85E-05 | 0.035 |
| *RPS26* | 738.11 | -1.95E-05 | 0.016 |
| *TCF3* | 242.87 | -1.42E-05 | 0.014 |
| *INSIG1* | 291.86 | -1.21E-05 | 0.032 |
| *ACSS2* | 127.61 | -1.09E-05 | 0.044 |
| *SAT1* | 223.40 | -1.04E-05 | 0.041 |
| *SPG7* | 163.79 | -9.93E-06 | 0.027 |
| *DDX42* | 197.64 | -9.17E-06 | 0.038 |
| *NPIPB5* | 128.30 | -7.90E-06 | 0.046 |
| *EIF3CL* | 71.00 | -6.30E-06 | 0.034 |
| *GOLGA8R* | 68.06 | -6.20E-06 | 0.032 |
| *FAM3C* | 56.18 | -5.63E-06 | 0.032 |
| *NPIPB3* | 54.11 | -5.45E-06 | 0.008 |
| *RPL17-C18orf32* | 31.61 | -4.59E-06 | 0.009 |
| *KLF7* | 13.85 | -3.14E-06 | 0.006 |
| *SMN1* | 20.02 | -2.91E-06 | 0.016 |
| *UBR3* | 41.88 | -2.38E-06 | 0.011 |
| *ZNF296* | 7.10 | -1.72E-06 | 0.025 |
| *NUDT4B* | 7.60 | -1.67E-06 | 0.034 |
| *INKA2* | 6.99 | -1.63E-06 | 0.019 |
| *GOLGA8O* | 35.76 | 9.25E-07 | 0.009 |
| *TPTEP2-CSNK1E* | 2.91 | 9.39E-07 | 0.022 |
| *PRICKLE4* | 4.41 | 1.46E-06 | 0.004 |
| *POC1B-GALNT4* | 6.36 | 1.81E-06 | 0.011 |
| *RPL36A-HNRNPH2* | 7.83 | 2.07E-06 | 0.011 |
| *ZNF490* | 9.36 | 2.10E-06 | 0.024 |
| *TRIM66* | 14.30 | 2.38E-06 | 0.023 |
| *WARS1* | 329.62 | 2.99E-06 | 0.019 |
| *USP40* | 27.62 | 3.60E-06 | 0.010 |
| *FAM72A* | 104.75 | 4.63E-06 | 0.017 |
| *MAGT1* | 28.08 | 4.96E-06 | 0.039 |
| *BAX* | 56.21 | 6.88E-06 | 0.012 |
| *AFTPH* | 115.71 | 7.18E-06 | 0.037 |
| *RPS10-NUDT3* | 111.26 | 7.58E-06 | 0.048 |
| *DVL3* | 113.30 | 8.07E-06 | 0.016 |
| *NIN* | 123.96 | 8.55E-06 | 0.012 |
| *NARF* | 122.19 | 9.62E-06 | 0.017 |
| *PPP1CB* | 309.10 | 1.17E-05 | 0.036 |
| *CCNA2* | 253.39 | 1.28E-05 | 0.022 |
| *PLK1* | 287.44 | 1.31E-05 | 0.029 |
| *HSD17B4* | 298.34 | 1.32E-05 | 0.028 |
| *CKAP2* | 501.50 | 1.81E-05 | 0.018 |
| *PSAT1* | 515.82 | 1.83E-05 | 0.017 |
| *PRKDC* | 1525.94 | 2.57E-05 | 0.034 |
| *FANCD2* | 142.19 | 3.42E-05 | 0.006 |
| *EIF3C* | 1076.07 | 0.20268 | 0.002 |
| *NUCB2* | 436.50 | 0.276024 | 0.004 |
| *AGAP5* | 9.46 | 4.13151 | 0.002 |
