# Supplementary material for: The synthetic opioid fentanyl increases HIV replication in macrophages
Source: PLoS One. 2025 Feb 27;20(2):e0298341. doi: 10.1371/journal.pone.0298341 (PMC11867328; doi:10.1371/journal.pone.0298341)
Supplement: S4 Table — (RTF) [file pone.0298341.s004.rtf]

S4 table.  GO enrichment for the differentially expressed genes usingToppGene in U937 cells infected with HIV in the presence / absence of fentanyl.

1: GO: Molecular Function

GO.ID	Name	pValue	FDR	FDR	Bonferroni	
			B&H	B&Y		
GO:0003735	structural constituent of ribosome	1.53E-04	3.90E-02	2.39E-01	3.90E-02	
GO:0070180	large ribosomal subunit rRNA binding	3.62E-04	4.62E-02	2.83E-01	9.24E-02	

2: GO: Biological Process
	
ID	NAME	PVALUE	FDR	FDR	BONFERRONI	
			B&H	B&Y		
GO:0002181	cytoplasmic translation	2.50E-07	4.76E-04	3.87E-03	4.76E-04	
GO:0043603	amide metabolic process	8.99E-06	3.92E-03	3.19E-02	1.71E-02	
GO:0140236	translation at presynapse	9.56E-06	3.92E-03	3.19E-02	1.82E-02	
GO:0140241	translation at synapse	1.03E-05	3.92E-03	3.19E-02	1.96E-02	
GO:0140242	translation at postsynapse	1.03E-05	3.92E-03	3.19E-02	1.96E-02	
GO:0006518	peptide metabolic process	1.49E-05	3.92E-03	3.19E-02	2.84E-02	
GO:0043604	amide biosynthetic process	1.60E-05	3.92E-03	3.19E-02	3.05E-02	
GO:0006412	translation	1.65E-05	3.92E-03	3.19E-02	3.13E-02	
GO:0043043	peptide biosynthetic process	2.32E-05	4.90E-03	3.99E-02	4.41E-02	
GO:0031109	microtubule polymerization or depolymerization	5.88E-05	1.12E-02	9.10E-02	1.12E-01	
GO:1903047	mitotic cell cycle process	7.51E-05	1.30E-02	1.06E-01	1.43E-01	
GO:0002326	B cell lineage commitment	9.27E-05	1.47E-02	1.20E-01	1.76E-01	
GO:0060028	convergent extension involved in axis elongation	1.86E-04	2.58E-02	2.10E-01	3.53E-01	
GO:0051098	regulation of binding	1.90E-04	2.58E-02	2.10E-01	3.62E-01	
GO:1902416	positive regulation of mRNA binding	2.76E-04	3.20E-02	2.60E-01	5.26E-01	
GO:0007020	microtubule nucleation	2.88E-04	3.20E-02	2.60E-01	5.48E-01	
GO:0000278	mitotic cell cycle	3.33E-04	3.20E-02	2.60E-01	6.35E-01	
GO:1905216	positive regulation of RNA binding	3.37E-04	3.20E-02	2.60E-01	6.42E-01	
GO:0033152	immunoglobulin V(D)J recombination	3.37E-04	3.20E-02	2.60E-01	6.42E-01	
GO:0022402	cell cycle process	3.38E-04	3.20E-02	2.60E-01	6.44E-01	
GO:0140013	meiotic nuclear division	3.59E-04	3.20E-02	2.60E-01	6.82E-01	
GO:0007051	spindle organization	3.94E-04	3.20E-02	2.60E-01	7.49E-01	
GO:0045132	meiotic chromosome segregation	4.03E-04	3.20E-02	2.60E-01	7.67E-01	
GO:1902415	regulation of mRNA binding	4.04E-04	3.20E-02	2.60E-01	7.69E-01	
GO:0010332	response to gamma radiation	4.89E-04	3.72E-02	3.03E-01	9.31E-01	
GO:0001933	negative regulation of protein phosphorylation	6.23E-04	4.51E-02	3.66E-01	1.00E+00	
GO:1905214	regulation of RNA binding	6.40E-04	4.51E-02	3.66E-01	1.00E+00	
GO:0001732	formation of cytoplasmic translation initiation complex	7.30E-04	4.96E-02	4.03E-01	1.00E+00	
3: GO: Cellular Component
				
ID	NAME	PVALUE	FDR	FDR	BONFERRONI	
			B&H	B&Y		
GO:0022626	cytosolic ribosome	1.63E-05	4.33E-03	2.67E-02	4.33E-03	
GO:0000137	Golgi cis cisterna	9.20E-05	8.18E-03	5.04E-02	2.45E-02	
GO:0005757	mitochondrial permeability transition pore complex	1.36E-04	8.18E-03	5.04E-02	3.63E-02	
GO:0000922	spindle pole	1.61E-04	8.18E-03	5.04E-02	4.27E-02	
GO:0044391	ribosomal subunit	1.61E-04	8.18E-03	5.04E-02	4.27E-02	
GO:0072686	mitotic spindle	1.85E-04	8.18E-03	5.04E-02	4.91E-02	
GO:0071541	eukaryotic translation initiation factor 3 complex, eIF3m	2.33E-04	8.85E-03	5.45E-02	6.19E-02	
GO:1990904	ribonucleoprotein complex	3.25E-04	1.08E-02	6.65E-02	8.64E-02	
GO:0031985	Golgi cisterna	4.57E-04	1.27E-02	7.83E-02	1.22E-01	
GO:0022625	cytosolic large ribosomal subunit	4.78E-04	1.27E-02	7.83E-02	1.27E-01	
GO:0005793	endoplasmic reticulum-Golgi intermediate compartment	6.72E-04	1.57E-02	9.67E-02	1.79E-01	
GO:0005840	ribosome	7.32E-04	1.57E-02	9.67E-02	1.95E-01	
GO:0005852	eukaryotic translation initiation factor 3 complex	7.67E-04	1.57E-02	9.67E-02	2.04E-01	
GO:0005925	focal adhesion	8.48E-04	1.61E-02	9.93E-02	2.26E-01	
GO:0033290	eukaryotic 48S preinitiation complex	9.75E-04	1.62E-02	1.00E-01	2.59E-01	
GO:0030055	cell-substrate junction	9.77E-04	1.62E-02	1.00E-01	2.60E-01	
GO:0016282	eukaryotic 43S preinitiation complex	1.09E-03	1.69E-02	1.04E-01	2.89E-01	
GO:0005795	Golgi stack	1.18E-03	1.69E-02	1.04E-01	3.13E-01	
GO:0070993	translation preinitiation complex	1.21E-03	1.69E-02	1.04E-01	3.21E-01	
GO:0005819	spindle	1.32E-03	1.76E-02	1.08E-01	3.52E-01	
GO:0005801	cis-Golgi network	1.43E-03	1.77E-02	1.09E-01	3.81E-01	
GO:0098556	cytoplasmic side of rough endoplasmic reticulum membrane	1.46E-03	1.77E-02	1.09E-01	3.89E-01	
GO:1990391	DNA repair complex	1.60E-03	1.81E-02	1.11E-01	4.25E-01	
GO:0005844	polysome	1.63E-03	1.81E-02	1.11E-01	4.34E-01	
GO:0033116	endoplasmic reticulum-Golgi intermediate compartment membrane	1.90E-03	2.03E-02	1.25E-01	5.07E-01	
GO:0032580	Golgi cisterna membrane	2.08E-03	2.13E-02	1.31E-01	5.54E-01	
GO:0097144	BAX complex	2.59E-03	2.55E-02	1.57E-01	6.89E-01	
GO:0099512	supramolecular fiber	2.89E-03	2.74E-02	1.69E-01	7.68E-01	
GO:0099081	supramolecular polymer	3.02E-03	2.77E-02	1.71E-01	8.04E-01	
GO:0031984	organelle subcompartment	3.20E-03	2.82E-02	1.74E-01	8.50E-01	
GO:0098554	cytoplasmic side of endoplasmic reticulum membrane	3.28E-03	2.82E-02	1.74E-01	8.73E-01	
GO:0015934	large ribosomal subunit	3.61E-03	2.97E-02	1.83E-01	9.59E-01	
GO:0046930	pore complex	3.69E-03	2.97E-02	1.83E-01	9.81E-01	
GO:0097145	BAK complex	5.16E-03	4.04E-02	2.49E-01	1.00E+00	
GO:0140534	endoplasmic reticulum protein- containing complex	5.56E-03	4.15E-02	2.56E-01	1.00E+00	
GO:0098791	Golgi apparatus subcompartment	5.62E-03	4.15E-02	2.56E-01	1.00E+00	
GO:0097431	mitotic spindle pole	5.77E-03	4.15E-02	2.56E-01	1.00E+00	
GO:0030867	rough endoplasmic reticulum membrane	6.03E-03	4.22E-02	2.60E-01	1.00E+00	
GO:0022627	cytosolic small ribosomal subunit	7.40E-03	4.78E-02	2.95E-01	1.00E+00	
GO:0099513	polymeric cytoskeletal fiber	7.47E-03	4.78E-02	2.95E-01	1.00E+00	
GO:0005745	m-AAA complex	7.73E-03	4.78E-02	2.95E-01	1.00E+00	
GO:0097124	cyclin A2-CDK2 complex	7.73E-03	4.78E-02	2.95E-01	1.00E+00	
GO:0032937	SREBP-SCAP-Insig complex	7.73E-03	4.78E-02	2.95E-01	1.00E+00	
