# Supplementary material for: The synthetic opioid fentanyl increases HIV replication in macrophages
Source: PLoS One. 2025 Feb 27;20(2):e0298341. doi: 10.1371/journal.pone.0298341 (PMC11867328; doi:10.1371/journal.pone.0298341)
Supplement: S2 Fig — On day 7, monocyte-derived macrophages [MDM] were harvested, and 1 x 10^5^ cells per well were plated. Fentanyl at 10 ug/mL was added and incubated for 24 hours. After 24 hours, cells were infected with HIVYK-JRCSF and incubated for 2 hours. The cells were rinsed three times to remove any unbound virus and replaced with fresh media with fentanyl and incubated for 3 days. Cells were harvested at the end of 72 hours and total RNA extraction was performed. Genes of interest were quantified by brilliant III ultrafast SYBR qRT-PCR. Error bars represent the standard deviations between replicates. Data were normalized to beta-globin expression and fold-change in expression was calculated by the 2 −ΔΔCT method. *p < 0.05; **p < 0.01; ***p < 0.001; ****p < 0.0001. (PDF) [file pone.0298341.s007.pdf]

Lorem Ipsum

Lorem ipsum dolor sit amet, consectetur adipiscing elit. Mauris maximus fringilla ligula, in malesuada erat tempor ac. Quisque dapibus posuere turpis, vel aliquam massa vehicula non.

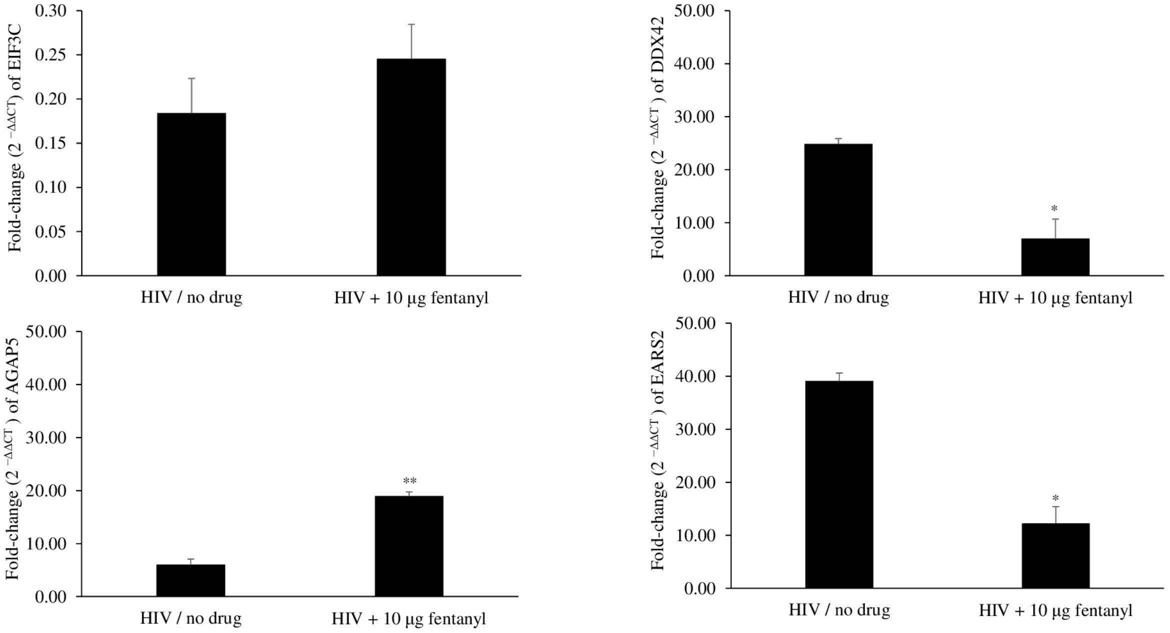

S2 figure. Primary monocytes at 1 x 10^5 cells were seeded in a flask and supplemented with RPMI 1640 + GM-CSF + IL-4 and incubated at 37°C for 6 days for monocyte to macrophage transformation. On day 7, monocyte derived macrophages [MDM] were harvested, and 1 x 10^5 cells per well were plated. Fentanyl at 10 ug/mL was added and incubated for 24 hours. After 24 hours, cells were infected with $HIV_{YK\text{-}JRCSF}$ and incubated for 2 hours. The cells were rinsed three times to remove any unbound virus and replaced with fresh media with fentanyl and incubated for 3 days. Cells were harvested at the end of 72 hours and total RNA extraction was performed. Genes of interest were quantified by brilliant III ultrafast SYBR qRT-PCR. Error bars represent the standard deviations between replicates. Data were normalized to Beta-globin expression and fold-change in expression was calculated by the $2^{-\Delta\Delta CT}$ method. *$p < 0.05$; **$p < 0.01$; ***$p < 0.001$; ****$p < 0.0001$.

test-test-1 This is a preview of your figure rendered on a simulated PLOS journal page.

Maecenas ac est sit amet odio sollicitudin euismod. In risus odio, convallis a neque ac, varius ultricies arcu. Vestibulum et quam iaculis, ultricies odio et, molestie magna. Suspendisse vehicula purus id turpis eleifend, et convallis dui dignissim. Praesent tempus elit a metus sollicitudin, sed fringilla nulla porttitor. Nullam in tempus massa. Nunc maximus magna massa, nec volutpat risus rhoncus ut. Fusce quis ante sem. Aenean nulla nibh, tempus sit amet rhoncus at, eleifend vel risus. Sed dictum, sem ultrices elementum pharetra, lacus diam volutpat orci, scelerisque semper dui lacus ut enim.

Suspendisse in nunc id lacus commodo consequat. Proin semper aliquam varius. Fusce vitae neque aliquam nisi ultrices sodales vitae ut enim. Vivamus nec dictum ipsum. Sed condimentum ante eu urna tincidunt tincidunt. In ac lacus nec ipsum viverra volutpat posuere vel lacus. Class aptent taciti sociosqu ad litora torquent per conubia nostra, per inceptos himenaeos. Morbi rhoncus ipsum quis lorem hendrerit, at vulputate massa tempus. Ut arcu nisl, gravida vitae risus ultricies, porta venenatis massa. Cras dignissim, enim at faucibus aliquam, sapien nisl eleifend dolor, vel mollis nulla nisi id ipsum. Pellentesque vehicula ultricies risus sit amet faucibus. Praesent sit amet mi ac est faucibus accumsan. Praesent pulvinar sit amet orci auctor feugiat.
